# PRMT5 inhibition induces pro‐inflammatory macrophage polarization and increased hepatic triglyceride levels without affecting atherosclerosis in mice

**DOI:** 10.1111/jcmm.17676

**Published:** 2023-03-22

**Authors:** Yiheng Zhang, Robin A. F. Verwilligen, Miranda Van Eck, Menno Hoekstra

**Affiliations:** ^1^ Division of BioTherapeutics, Leiden Academic Centre for Drug Research (LACDR) Leiden University Leiden The Netherlands; ^2^ Division of Systems Pharmacology and Pharmacy, Leiden Academic Centre for Drug Research (LACDR) Leiden University Leiden The Netherlands; ^3^ Pharmacy Leiden Leiden The Netherlands

**Keywords:** atherosclerosis, fatty liver disease, inflammation, macrophages, protein arginine methyltransferase 5

## Abstract

Protein arginine methyltransferase 5 (PRMT5) controls inflammation and metabolism through modulation of histone methylation and gene transcription. Given the important role of inflammation and metabolism in atherosclerotic cardiovascular disease, here we examined the role of PRMT5 in atherosclerosis using the specific PRMT5 inhibitor GSK3326595. Cultured thioglycollate‐elicited peritoneal macrophages were exposed to GSK3326595 or DMSO control and stimulated with either 1 ng/mL LPS or 100 ng/mL interferon‐gamma for 24 h. Furthermore, male low‐density lipoprotein (LDL) receptor knockout mice were fed an atherogenic Western‐type diet and injected intraperitoneally 3×/week with a low dose of 5 mg/kg GSK3326595 or solvent control for 9 weeks. In vitro, GSK3326595 primed peritoneal macrophages to interferon‐gamma‐induced M1 polarization, as evidenced by an increased M1/M2 gene marker ratio. In contrast, no difference was found in the protein expression of iNOS (M1 marker) and ARG1 (M2 marker) in peritoneal macrophages of GSK3326595‐treated mice. Also no change in the T cell activation state or the susceptibility to atherosclerosis was detected. However, chronic GSK3326595 treatment did activate genes involved in hepatic fatty acid acquisition, i.e. SREBF1, FASN, and CD36 (+59%, +124%, and +67%, respectively; *p* < 0.05) and significantly increased hepatic triglyceride levels (+50%; *p* < 0.05). PRMT5 inhibition by low‐dose GSK3326595 treatment does not affect the inflammatory state or atherosclerosis susceptibility of Western‐type diet‐fed LDL receptor knockout mice, while it induces hepatic triglyceride accumulation. Severe side effects in liver, i.e. development of non‐alcoholic fatty liver disease, should thus be taken into account upon chronic treatment with this PRMT5 inhibitor.

## 1 INTRODUCTION

The type II protein arginine methyltransferase (PRMT) family member PRMT5 controls cell proliferation, inflammation, and metabolism through modulation of histone methylation and gene transcription.^1^ Due to its central role in these essential processes, PRMT5 has been associated with a variety of diseases. As reviewed by Xiao et al., PRMT5 is highly expressed in different types of cancer where it contributes to enhanced tumour cell proliferation and invasion.^2^ In addition, PRMT5 protects the HIV‐1 accessory protein viral protein R from proteasomal degradation to support HIV replication^3^ and regulates the secretion of pro‐inflammatory cytokines such as interleukin‐6 (IL‐6) and interferon (IFN)‐gamma inducible protein‐10 (IP‐10/CXCL10) by macrophages in endometriosis.^4^ Furthermore, Webb et al. have shown that PRMT5 is upregulated upon memory T cell reactivation and essential for T cell activation and expansion in delayed‐type hypersensitivity and experimental autoimmune encephalomyelitis.^5^

Interestingly, studies by Tan et al. have suggested that changes in PRMT5 functionality may also be relevant in the context of atherosclerotic cardiovascular disease, a progressive pathology that includes macrophage‐driven inflammation secondary to dyslipidemia‐induced cholesterol accumulation within the arterial wall.^6^ More specifically, Tan et al. showed that the relative mRNA expression levels of PRMT5 in peripheral blood cells are reduced with increasing coronary artery disease severity.^7^ We therefore hypothesize that a potential causal relationship may exist between PRMT5 activity, inflammation, and atherosclerosis development. To test this hypothesis, in the current study we determined the effect of treatment with the selective PRMT5 inhibitor GSK3326595 on (1) the macrophage response to pro‐inflammatory stimuli in vitro and ex vivo and (2) atherosclerosis development in hypercholesterolemic low‐density lipoprotein (LDL) receptor knockout mice in vivo.

## 2 MATERIALS AND METHODS

### 2.1 In vitro experiment with thioglycollate‐elicited peritoneal macrophages

C57BL/6 Three C57BL/6 wild‐type female mice received an intraperitoneal injection with 1 mL of 3% thioglycollate to elicit macrophage recruitment to the peritoneal cavity. After 5 days, mice were sacrificed and the peritoneal cavity flushed with PBS. The collected peritoneal macrophages were pooled together and plated in 12‐well plates at a concentration of one million cells per ml in Dulbecco's Modified Eagle Medium (DMEM), containing 10% fetal bovine serum, penicillin/streptomycin and L‐glutamine. After allowing the cells to attach to the culture plate for 4 h (5% CO_2_ and 36°C), cells were washed with PBS, and exposed to 0.1% DMSO or 100 μM GSK3326595 dissolved in 0.1 DMSO% and in the presence of PBS as control, 1 ng/mL LPS (L3024‐5MG, Merck), or 100 ng/mL IFN‐gamma (I4777‐1MG, Merck). Each condition was tested in 6 independent wells (*n* = 6 replicates). After 12 h, cell media were collected for cytokine detection by ELISA. Cells were lysed in guanidine thiocyanate (GTC) (L‐15809, Fisher Scientific) for further mRNA expression analysis.

### 2.2 Gene expression analysis through real‐time quantitative PCR

Total RNA was isolated according to Chomczynski and Sacchi.^8^ The concentration of the obtained RNA was determined using a Nanodrop Spectrophotometer (Nanodrop Technologies). cDNA was synthesized from the RNA using Maxima H Minus reverse transcriptase (Thermo Scientific, Cat. no. EP0751). Analysis of gene expression was accomplished through an ABI PRISM 7500 machine (Applied Biosystems) using SYBR Green technology. Β‐actin, ribosomal protein L27 (RPL27), acidic ribosomal phosphoprotein P0 (36B4) and peptidylpropyl isomerase A (PPIA) were used as housekeeping genes. The sequences of the primers used can be found in Table 1. The relative gene expression was calculated by subtracting the threshold cycle number (Ct) of the genes of interest from the average housekeeping Ct values and raising 2 to the power of this difference. To exclude variations in the relative gene expression of housekeeping genes, the average Ct values of the four housekeeping genes were used. For detailed information about the protocol, see the materials and methods in Appendix S1.

**TABLE 1:** Nucleotide sequences of primers used for RT‐PCR.

Gene	Genbank accession no.	Forward primer	Reverse primer
PPIA	NM_008907.2	AGGATTTGGCTATAAGGGTTCCTCC	ATTTCTCTCCGTAGATGGACCTGC
RPL27	NM_011289.3	CGCCAAGCGATCCAAGATCAAGTCC	AGCTGGGTCCCTGAACACATCCTTG
36B4	NM_007475.5	CTGAGTACACCTTCCCACTTACTGA	CGACTCTTCCTTTGCTTCAGCTTT
TNF‐alpha	NM_013693.3	CAAAGGGATGAGAAGTTCCCAAATGGC	CACTCCAGCTGCTCCTCCACTTG
IL‐1beta	NM_008361.4	AACGACAAAATACCTGTGGCCTTG	CCGTTTTTCCATCTTCTTCTTTGGGT
MHCII	NM_010378.3	TCTGTGGAGGTGAAGACGACATTGAG	TCAAATGTGTACTGGCCAATGTCTCCA
IP‐10 (CXCL10)	NM_021274.2	GTCATTTTCTGCCTCATCCTGCTG	CCTATGGCCCTCATTCTCACTGG
iNOS	NM_010927.4	TCTGCAGCACTTGGATCAGGAACCT	AGAAACTTCGGAAGGGAGCAATGCC
ARG1	NM_007482.3	TGGCAGAGGTCCAGAAGAATGG	GTGAGCATCCACCCAAATGACAC
CD206 (MRC1)	NM_008625.2	ACGGATAGATGGAGGGTGCGGT	GCAGTTGAGGAGGTTCAGTAGCAGG
CD68	NM_001291058.1	TTGACCTGCTCTCTCTAAGGCTACAG	AGGACCAGGCCAATGATGAGAGG
CCL2	NM_011333.3	CTGAAGCCAGCTCTCTCTTCCTC	GGTGAATGAGTAGCAGCAGGTGA
APOB	NM_009693.2	GGAGTTAAACATTTTATCAAACATAGGCCAACA	AACTCTCTCCAAAAAGCCCTGAAGAC
MTTP	NM_001163457.2	TCTCACAGTACCCGTTCTTGGT	GAGAGACATATCCCCTGCCTGT
LIPE	NM_010719.5	GCCTACTGCTGGGCTGTCAAGC	AGGGACACAGTGATGCAGAGATTCCC
ATGL	NM_001163689.1	GCCAACGCCACTCACATCTACGG	GAGGGATGCAGAGGACCCAGGAAC
PNPL3	NM_054088.3	GAGTCGGCTATCGCTGCAGTC	GCCCATTGTAGCCACTGGATATCATC
ACOX1	NM_015729.3	GCCAAGAAGTCCCCACTGAACAAGAC	GGGAAACTTCAAAGCTTCGACTGCAG
CPT1	NM_013495.2	GGTTGCTGATGACGGCTATGGT	TGGCTTGTCTCAAGTGCTTCCC
PPARα	NM_011144.6	TGACATTTCCCTGTTTGTGGCTGCT	TGCACAATCCCCTCCTGCAACTTC
PGC1α	NM_008904.2	CGCTTTCGCTGCTCTTGAGAATGGA	TGGTGGAAGCAGGGTCAAAATCGTC
CD36	NM_001159558.1	ATGGTAGAGATGGCCTTACTTGGG	AGATGTAGCCAGTGTATATGTAGGCTC
SREBF1	NM_011480.4	TCTGAGGAGGAGGGCAGGTTCCA	GGAAGGCAGGGGGCAGATAGCA
FASN	NM_007988.3	GGCGGCACCTATGGCGAGG	CTCCAGCAGTGTGCGGTGGTC
SCD1	NM_009127.4	GGAAAGTGAGGCGAGCAACTGACTA	CAGGACGGATGTCTTCTTCCAGGTG
ACACA	NM_133360.2	GGAAGATGGCGTCCGCTCTGTG	GTGAGATGTGCTGGGTCATGTGGAC

### 2.3 Cytokine enzyme‐linked immunosorbent assay (ELISA)

For measurement of the concentrations of the pro‐inflammatory cytokine IP‐10 in the cell culture medium, we used the Mouse CXCL10/IP‐10/CRG‐2 DuoSet ELISA kit (Cat. No. DY466‐0) from R&D systems. Absorbances were measured at 450 nm and 570 nm.

### 2.4 Experimental animals

Ten‐week‐old male LDL receptor knockout mice (on a C57BL6/J background), obtained from The Jackson Laboratory and bred at Gorlaeus Laboratories, were fed a Western‐type diet containing 0.25% cholesterol and 15% cocoa butter (SDS, Sussex, UK) to induce the development of atherosclerotic lesions. The mice were randomly allocated to two different treatment groups receiving intraperitoneal injections of either the control solvent DMSO (100 μL 10% DMSO in PBS, *N* = 12) or GSK3326595 (0.125 mg in 100 μL 10% DMSO in PBS, ~5 mg/kg based on body weight at start of the experiment, *N* = 15) three times per week for altogether 9 weeks. As Gerhart et al. showed that an in vivo treatment dosage of 4.2 mg/kg GSK3326595 is able to reduce the synthesis of the PRMT5 product symmetric dimethylarginine by >80%,^9^ we chose 5 mg/kg as our treatment dosage. For sacrifice, mice were anaesthetised through a subcutaneous injection with 100–150 μL of a ketamine (100 mg/kg), xylazine (12.5 mg/kg), and atropine (125 μg/kg) mixture. Subsequently, orbital blood was collected and a whole‐body perfusion was performed using PBS. Organs were excised and parts were fixed for 24 h in a 3.7% formalin solution for subsequent histological analysis or stored at −20°C for biochemical analysis.

All experimental protocols were approved by the Animal Welfare Body of Leiden University under the project licence AVD1060020185964 issued by the Central Authority for Scientific Procedures on Animals (CCD). The study was executed according to the principles of laboratory animal care and regulations of Dutch law on animal welfare, the Directive 2010/63/EU of the European Union, and the ARRIVE guidelines.

### 2.5 Ex vivo experiment with peritoneal macrophages

Upon sacrifice of the LDL receptor knockout mice, peritoneal cells were collected from both the DMSO and GSK3326595‐treated groups. These were subsequently plated in 12‐well plates in DMEM, containing 10% fetal bovine serum, penicillin/streptomycin, and L‐glutamine. After allowing the cells to attach to the culture plate for 4 h at 5% CO_2_ and 37°C, cells were exposed to PBS as control or to 100 ng/mL IFN‐gamma for ex vivo activation. After 12 h, cell medium was collected for cytokine detection by ELISA and cells were lysed in GTC for further mRNA expression analysis.

### 2.6 Flow cytometry

Immunostaining was performed on single cell suspensions derived from the blood, spleen, and peritoneum of LDL receptor knockout mice treated with GSK3326595 or DMSO for 9 weeks. Single cell suspensions were obtained by filtering the samples/organs through a 70 μm cell strainer using PBS. Cells were stained using the appropriate fluorochrome‐labelled antibodies (Table 2). To select the living cells, Fixable Viability Dye eFluorTM 780 (1:2000, eBioscience) was used. Unconjugated anti‐CD16/32 antibody (clone 2.4G2, BD Bioscience) was applied to block Fc receptors. For intracellular staining, cells were fixed and permeabilized by using transcription factors fixation/permeabilization concentrate and diluent solution (BD Biosciences). The flow cytometry gating strategy for CD4+ and CD8+ T cells in the blood, peritoneal cavity, and spleen is shown in the Appendix S1 (Figure S1). Flow cytometric analysis was performed on a Beckman Coulter Cytoflex S and the acquired data were analysed using FlowJo software.

**TABLE 2:** Flow cytometry antibodies.

	Fluorochrome	Clone	Supplier
Extracellular markers			
CD4	PercP	RM4‐5	BD biosciences
CD8	BV510	53–6.7	Biolegend
CD11b	BV605	M1/70	Biolegend
CD44	APC	IM7	eBioscience
CD45	PE	30‐F11	Biolegend
CD62L	BV605	MEL14	Biolegend
F4/80	FITC	BM8	Biolegend
Intracellular markers			
Arg1	AF700	AlexF5	eBioscience
iNOS	PE‐dazzle 594	W16030C	Biolegend

### 2.7 Atherosclerotic lesion histological analysis

At sacrifice, hearts were embedded in OCT compound (Optimum Cutting Temperature; Sakura Finetek Europe B.V., Alphen aan den Rijn, The Netherlands) and stored at −80°C. Cryosections of 10 μm of the aortic root were collected on Menzel Gläser SuperFrost® Plus slides (Thermo Scientific; USA, Cat. no. 631–9483). Collection of aortic root coupes started at the appearance of the 3 valves and 9–10 sections per slide were collected, sectioned at a thickness of 10 μm (80 μm interval). Sections were routinely stained for the presence of neutral lipids using Oil red O and Gills no. 3 haematoxylin (GH380, Sigma‐Aldrich, Zwijndrecht, The Netherlands). For identification of macrophages in sections containing atherosclerotic lesions, a primary rat anti‐mouse monoclonal MOMA‐2 antibody (MCA519G; Bio‐Rad) was used at a 1:1000 dilution in blocking buffer. A secondary alkaline phosphatase‐conjugated goat‐anti‐rat IgG (A8438, Sigma‐Aldrich, Zwijndrecht, The Netherlands) was used at a dilution of 1:100 in blocking buffer, followed by the Vectastain ABC kit (PK‐4000, Vector laboratories, USA). ImmPACT NovaRED HRP (SK‐4800, Vector laboratories) as an enzyme substrate for signal detection. Sections were stained using Masson's Trichrome (Sigma‐Aldrich) to show the presence of collagen. Mean atherosclerotic lesion areas, macrophage areas, and collagen‐rich areas (in μm^2^) were quantified using pictures generated with a digital slide scanner (PANNORAMIC 250 Flash II, 3dHistech) and image J software. The mean of three different sections (number of repeats) is shown as one measurement per mouse. Quantification was performed blinded and using ImageJ.

### 2.8 Plasma lipid measurements

Plasma specimens from LDL receptor mice were isolated from orbital blood samples and collected in K2 EDTA tubes (MiniCollect Tube 450,532,Greiner Bio‐One). After 10 min of centrifugation at 8000 rpm (6082 rcf), plasma concentrations of free cholesterol, cholesterol esters, and triglycerides were determined using enzymatic colorimetric assays (Roche Diagnostics). For each colorimetric assay, precipath (PreciControl ClinChem Multi 2, cat no. 05947774) was used as internal calibrator.

### 2.9 Histological staining

Brown adipose tissue (BAT), white adipose tissue (WAT), and liver tissues were fixed in 1:4 formalin (Formal‐fix; Shandon Scientific Ltd., UK; cat. no. 9990245) for 24 h and stored in 0.1% sodium azide in PBS. BAT and WAT were embedded in paraffin, sectioned at 5 μm using a microtome, and stained with Eosin–Haematoxylin (Sigma‐Aldrich, Zwijndrecht, The Netherlands).

Liver tissues were embedded in Tissue‐Tek® O.C.T. Compound (Sakura Finetek Europe, cat no. 4583, Alphen aan den Rijn, The Netherlands) and 10 μm thick cryosections were prepared. Liver cryosectons were stained for the presence of neutral lipids using Oil Red O and haematoxylin (Sigma‐Aldrich, Zwijndrecht, The Netherlands). Representative photomicrographs were taken for tissues from each group.

### 2.10 Tissue lipid extraction and quantification

Tissue lipids were extracted from ~50 mg of BAT, WAT, and liver per mouse. For lipid extraction, the tissue pieces were homogenized in 500 μL PBS. For triglyceride extraction, the tissue pieces were homogenized in 500 μL 5% Nonidet™ P 40 Substitute. The solution was heated to 90°C and cooled on ice for 2 min each. Subsequently, insoluble material was removed from the solution through centrifugation and triglycerides in the supernatant were measured using an enzymatic colorimetric assay (Roche Diagnostics). Cholesterol was extracted using the protocol of Bligh and Dyer^10^ and quantified using an enzymatic colorimetric assay (Roche Diagnostics). For each colorimetric assay, precipath (Roche Diagnostics) was used as internal calibrator. Protein quantification was performed using the Pierce™ BCA protein assay Kit (ThermoFisher, Cat. no 23225) following manufacturer's instruction. The concentration of triglycerides and cholesterol in liver samples was corrected for the amount of tissue protein and expressed as μg lipid / mg protein.

### 2.11 Statistical analysis

Statistical analysis was performed using GraphPad Prism 8.0 (GraphPad Software Inc.). A Grubbs' test was used to test for outliers within groups. Two‐tailed unpaired Student's *t*‐test was used to calculate the significance of differences between single groups. A *p*‐value below 0.05 was considered statistically significant for all tests. Data are presented in graphs as means ± SEM or individual points with respective group averages.

## 3 RESULTS

### 3.1 PRMT5 inhibition by GSK3326595 primes peritoneal macrophages to IFN‐gamma‐induced M1 polarization in vitro

**FIGURE 1:**
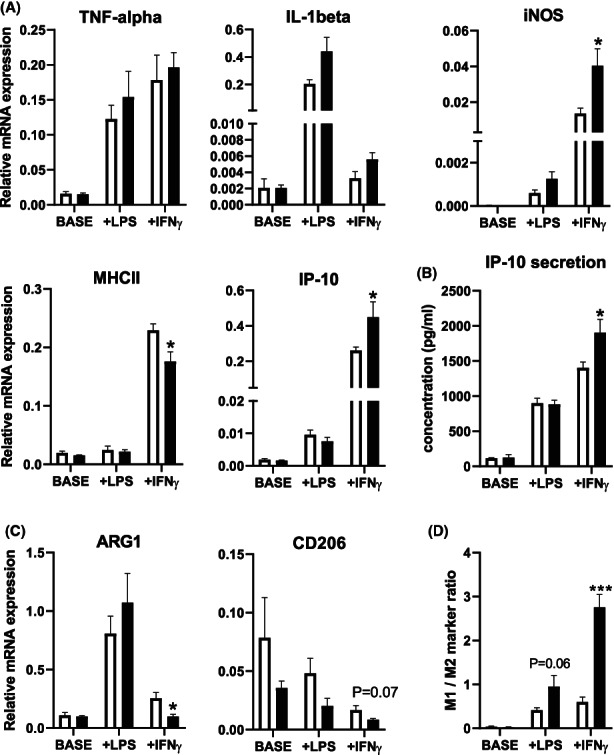
PRMT5 inhibition by GSK3326595 primes peritoneal macrophages to IFN‐gamma‐induced M1 polarization in vitro. (A) Relative mRNA expression levels of M1 markers TNF‐alpha, IL‐1beta, INOS, MHCII and IP‐10 at baseline (Base; non‐stimulated) or in response to LPS or IFN‐gamma. (B) The pro‐inflammatory cytokine IP‐10 protein secretion levels in thioglycollate‐elicited peritoneal macrophages treated with DMSO as control group (DMSO; white) or 100 μM GSK3326595 (GSK; black) for 12 h with/without stimulation with either 1 ng/mL LPS or 100 ng/mL IFN‐gamma. (C) Relative mRNA expression levels of M2 markers ARG1 and CD206 and (D) M1/M2 ratio. Each condition was tested in six independent wells of pooled peritoneal cells isolated from three mice (*N* = 6 replicates). Data are expressed as means ± SEM. *p < 0.05, ***p < 0.001 (Two‐tailed unpaired Student's *t*‐test).

Studies in mouse RAW264 and human THP‐1 macrophage cell lines have suggested that PRMT5 is involved in the cellular response to pro‐inflammatory stimuli such as lipopolysaccharide (LPS) and IFN‐gamma.^4^, ^11^ To provide additional insight into the contribution of PRMT5 to macrophage inflammation, cultured thioglycollate‐elicited mouse peritoneal macrophages were treated with 100 μM PRMT5 inhibitor GSK3326595 or a similar volume of the solvent control DMSO for 24 h in parallel with exposure to LPS or IFN‐gamma. In accordance with an immuno‐resolving role of thioglycollate‐elicited peritoneal macrophages,^12^ the cultured cells did not exhibit a pro‐inflammatory phenotype in the basal state as judged from the observation that relative mRNA expression levels of pro‐inflammatory M1 macrophage gene markers TNF‐alpha, iNOS, IL‐1beta, MHC‐II, and IP‐10 (Figure 1A) were on average lower than those of the anti‐inflammatory/resolving macrophage marker genes ARG1 and CD206 (Figure 1C). Treatment with GSK3326595 for 24 h was not associated with a change in the baseline macrophage phenotype. As expected, subsequent exposure of the thioglycollate‐elicited macrophages to LPS and IFN‐gamma induced a shift in the M1/M2 gene marker ratio towards a more pro‐inflammatory phenotype characterized by high TNF‐alpha, iNOS, and IP‐10 expression levels. Importantly, relative gene expression levels of iNOS (+207%, *p* < 0.05) and IP‐10 (+73%, *p* < 0.05) were even higher in cells pre‐treated with GSK3326595 than in those treated with solvent control after IFN‐gamma stimulation, but not after LPS exposure (Figure 1A). As a result, GSK3326595 treatment was associated with a greater rise in the overall M1/M2 gene marker ratio (*p* < 0.001; Figure 1D) and IP‐10 protein secretion (+37%, *p* < 0.05, Figure 1B **)** in response to the IFN‐gamma challenge as compared to DMSO control group. These combined findings imply that PRMT5 inhibition generates macrophages more susceptible to obtain a pro‐inflammatory phenotype specifically in the presence of IFN‐gamma. Notably, pharmacological PRMT5 inhibition was associated with a diminished IFN‐gamma‐induced increase in relative mRNA expression levels of MHCII (−23%; *p* < 0.05). It thus appears that IFN‐gamma driven M1 pro‐inflammatory macrophage polarization and MHCII expression induction are uncoupled through PRMT5 inhibitor pre‐treatment.

### 3.2 Treatment of LDL receptor knockout mice with GSK3326595 is associated with exacerbated IFN‐gamma‐induced M1 macrophage polarization ex vivo

**FIGURE 2:**
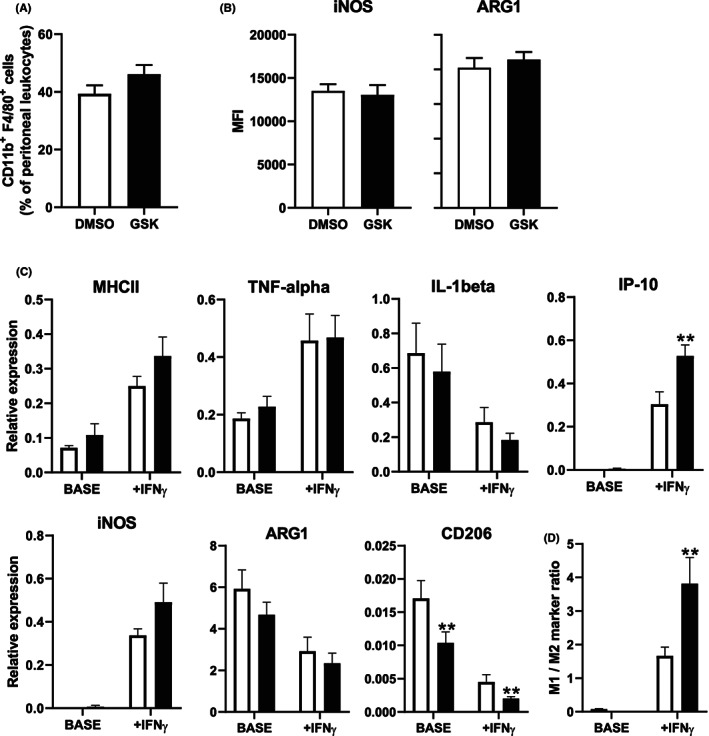
Treatment of LDL receptor knockout mice with GSK3326595 is associated with exacerbated IFN‐gamma‐induced M1 macrophage polarization ex vivo. (A) Relative numbers of CD11b^+^F4/80^+^ macrophages and (B) their mean fluorescent intensities (MFI) of the pro‐inflammatory M1 marker INOS and the anti‐inflammatory M2 marker ARG1 within the peritoneal cell population of male LDL receptor knockout mice treated either with DMSO solvent control (white, *N* = 12) or 5 mg/kg GSK3326595 (black, *N* = 15). (C) Relative mRNA expression levels of M1 or M2 macrophage marker genes and (D) the relative M1/M2 expression ratio in non‐simulated (Base) and in 100 ng/mL IFN‐gamma ex vivo stimulated peritoneal macrophages. Data are expressed as means ± SEM. **p < 0.01 (Two‐tailed unpaired Student's *t*‐test).

To determine the effect of GSK3326595 treatment on macrophage polarization and atherosclerosis susceptibility in vivo, atherosclerosis‐susceptible hypercholesterolemic LDL receptor knockout mice were intraperitoneally injected three times per week with 5 mg/kg GSK3326595 or a similar volume of the solvent control (100 μL 10% DMSO in PBS) while being fed a Western‐type diet enriched in cholesterol and fat for 9 weeks. Peritoneal cells were collected to investigate the effect of PRMT5 inhibition on the baseline phenotype of resident macrophages and the ability of IFN‐gamma to induce M1 macrophage polarization ex vivo. Flow cytometric analysis suggested that chronic GSK3326595 treatment did not change the percentage of peritoneal macrophages (Figure 2A) or their basal iNOS and ARG1 protein expression levels (Figure 2B). Accordingly, although CD206 mRNA expression levels were reduced by GSK3326595 treatment (−39%; *p* < 0.01; Figure 2C), gene expression levels of other M2 marker genes as well as M1 markers were, in general, not different between the cultured peritoneal macrophage populations (Figure 2C). However, peritoneal macrophages isolated from the GSK3326595‐treated mice did display an exacerbated polarization response upon exposure to IFN‐gamma, resulting in a 2.3‐fold higher M1/M2 ratio (*p* < 0.01; Figure 2D). This latter effect could be attributed to elevated mRNA expression levels of IP‐10 (+73%; *p* < 0.01), in line with our in vitro findings, as well as a reduction in CD206 transcript levels (−41%; *p* < 0.01) (Figure 2C).

### 3.3 Treatment of LDL receptor knockout mice with GSK3326595 does not change T cell numbers or phenotype

**FIGURE 3:**
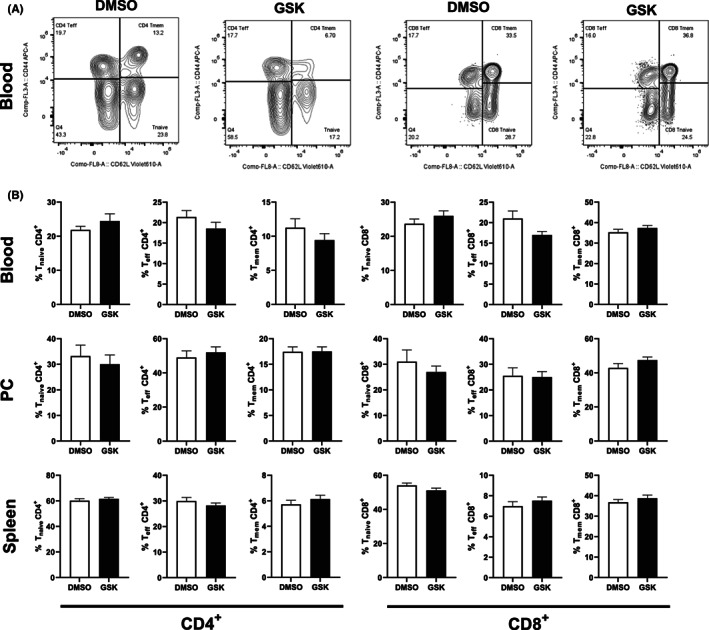
Treatment of LDL receptor knockout mice with GSK3326595 does not affect the T cell phenotype. (A) Representative flow cytometry plots of CD4+ and CD8+ CD44 and CD62L. (B) Distribution of CD4+ and CD8+ T cells over their respective naïve (CD44−, CD62L+), effector (CD44+, CD62L−), and memory cell (CD44+, CD62L+) subsets in the blood (top row), peritoneal cavity (middle row), and spleen (bottom row) from male LDL receptor knockout mice treated either with DMSO solvent control (white, *N* = 12) or 5 mg/kg GSK3326595 (black, *N* = 15). Data are expressed as means ± SEM (Two‐tailed unpaired Student's *t*‐test).

Given that PRMT5 has also been suggested to play a role in T cell differentiation and survival^5^, ^13^, ^14^ a potential effect of our chronic GSK3326595 treatment on T cell subsets was examined by means of flow cytometry. PRMT5 inhibition did not significantly impact total CD4^+^ helper and CD8^+^ cytotoxic T cell numbers in any of the compartments studied (data not shown). Staining for the cell surface markers CD62L and CD44 revealed that both the CD4^+^ and CD8^+^ T cells were also distributed equally between the two treatment groups over their respective naïve (CD44^−^CD62L^+^), effector (CD44^+^CD62L^−^), and memory (CD44^+^CD62L^+^) subclasses (Figure 3).

### 3.4 Treatment of LDL receptor knockout mice with GSK3326595 does not alter atherosclerosis susceptibility

**FIGURE 4:**
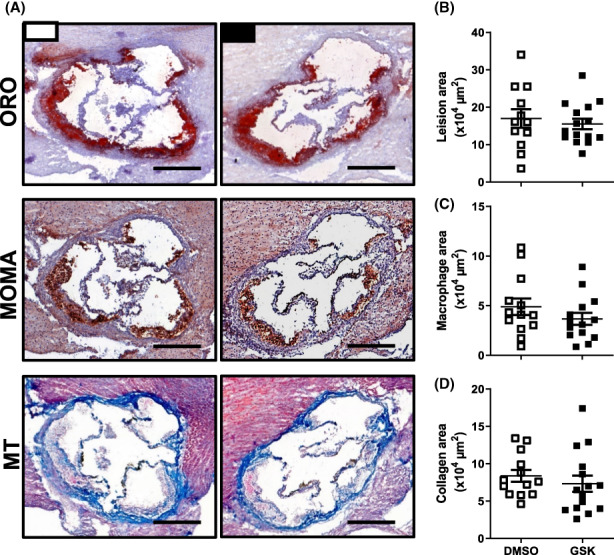
Treatment of LDL receptor knockout mice with GSK3326595 does not alter atherosclerosis susceptibility. (A) Representative pictures of aortic root cryosections from male LDL receptor knockout mice treated DMSO solvent control (white, *N* = 12) or 5 mg/kg GSK3326595 (black, *N* = 15) stained with Oil red O (ORO; neutral lipids in red), MOMA (macrophages in brown) or Masson's Trichrome (MT; collagen in blue) and the quantification of (B) total lesion area (C) lesional macrophage area and (D) the plaque collagen content. Scale bar = 200 μm. The mean of three different sections per mouse (number of repeats) is depicted as one data point per mouse. Data are shown as individual points with the group average as horizontal lines (Two‐tailed unpaired Student's *t*‐test).

Sections of the aortic root were stained with Oil red O for neutral lipids to identify atherosclerotic lesions. As can be seen from the representative images in Figure 4A, Oil red O‐positive lesions were present in both GSK3326595‐treated and solvent control‐treated mice after 9 weeks of Western‐type diet feeding. Quantification of total Oil red O‐positive areas revealed that PRMT5 inhibition did not significantly change the atherosclerosis susceptibility. GSK3326595‐treated mice developed lesions with an average size of 16 ± 1 × 10^4^ μm^2^, whilst average lesion sizes of control mice were 17 ± 2 × 10^4^ μm^2^ (Figure 4B). MOMA‐2 staining showed that lesions of both groups of mice were equally rich in macrophages (4 ± 1 × 10^4^ μm^2^ for GSK3326595‐treated mice versus 5 ± 1 × 10^4^ μm^2^ for control‐treated mice; Figure 4C). Trichrome staining further verified a similar lesion composition, with collagen areas of ~7 × 10^4^ μm^2^ in both groups of mice (Figure 4D).

### 3.5 GSK2236595 treatment increases hepatic triglyceride levels without changing the hyperlipidemia extent in LDL receptor knockout mice

**FIGURE 5:**
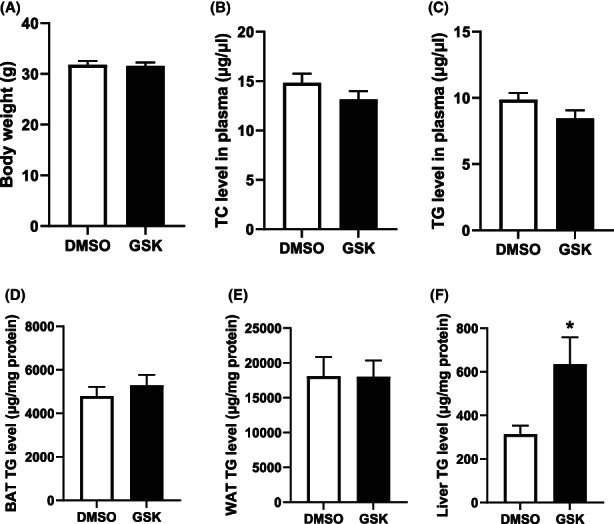
GSK2236595 treatment increases hepatic triglyceride levels without changing the hyperlipidemia extent in LDL receptor knockout mice. (A) Body weights and (B) plasma total cholesterol (TC) and (C) triglyceride (TG) levels from Western‐type diet‐fed male LDL receptor knockout mice treated DMSO solvent control (white, *N* = 12) or 5 mg/kg GSK3326595 (black, *N* = 15). Tissue triglyceride (TG) levels in (D) BAT, (E) WAT and (F) liver. Data are expressed as means ± SEM. *p < 0.05 (Two‐tailed unpaired Student's *t*‐test).

**FIGURE 6:**
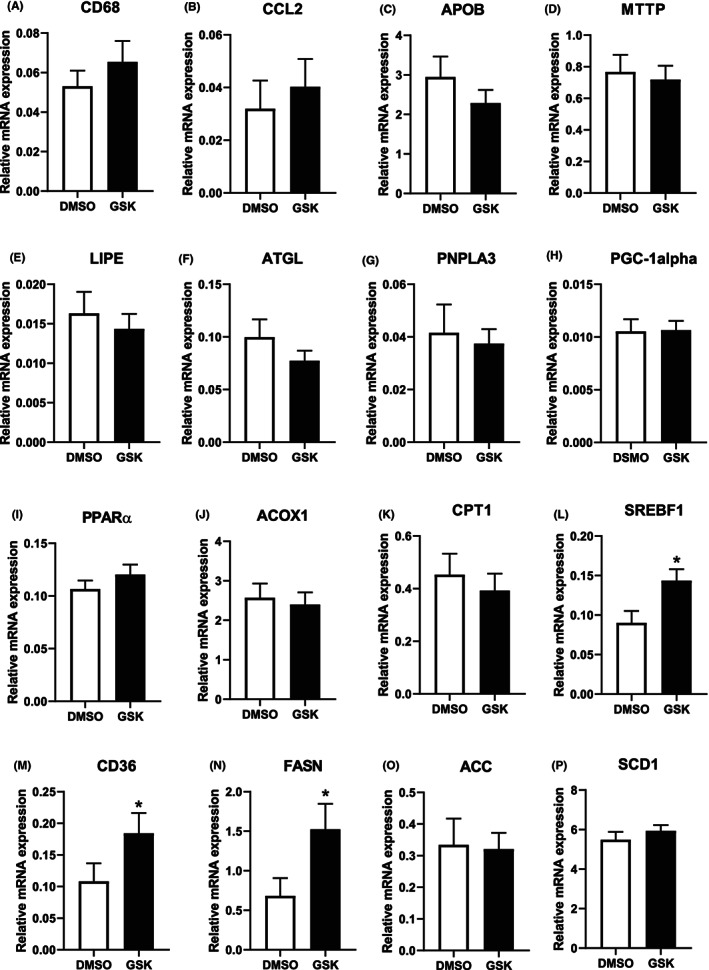
GSK2236595 treatment activates genes involved in fatty acid acquisition in livers of LDL receptor knockout mice. Relative mRNA expression levels in livers from male LDL receptor knockout mice treated DMSO solvent control (white, *N* = 12) or 5 mg/kg GSK3326595 (black, *N* = 15) of genes involved in liver inflammation (A, B), hepatic VLDL synthesis (C, D), lipolysis and fatty acid oxidation (E–K), and fatty acid synthesis and acquisition (L–P). Values are relative to the housekeeping expression levels and expressed as means ± SEM. *p < 0.05 (Two‐tailed unpaired Student's *t*‐test).

Liu et al. have shown that PRMT5 can enhance the activity of the lipogenic transcription factor SREBP‐1a.^15^ In addition, recent findings by Wang et al. have suggested that PRMT5 is involved in the control of LDL uptake by hepatocytes.^16^ Given that disturbances in lipid metabolism are the driving force in the initiation of atherosclerotic lesion development, we investigated a potential effect of GSK3326595 treatment on the body's lipid status. Weight development was not influenced by GSK3326595 treatment (data not shown). As a result, average body weights at sacrifice, after 9 weeks of Western‐type diet feeding, were not significantly different between the two treatment groups (Figure 5A). In accordance with a similar body weight development, no differences were found in the triglyceride content of perigonadal white adipose tissue and subcutaneous brown fat depots (Figure 5D–F).^17^, ^18^ Strikingly, an on average 50% higher (*p* < 0.05) hepatic triglyceride content was detected in GSK3326595‐treated mice as compared to control‐treated mice (Figure 5D–G). The apparent change in fatty liver development upon the Western‐type diet challenge was not associated with an altered inflammation state of the liver as judged from the unchanged hepatic relative mRNA expression levels of the general macrophage marker CD68 and the macrophage‐derived cytokine CCL2 (Figure 6A,B). Furthermore, no concomitant change in the hyperlipidemia extent, that is plasma total cholesterol and triglyceride levels, was observed (Figure 5B,C).

### 3.6 GSK2236595 treatment activates genes involved in fatty acid acquisition in livers of LDL receptor knockout mice

Gene expression analysis on liver specimens was performed to potentially uncover the mechanism behind the rise in hepatic triglyceride content in response to pharmacological PRMT5 activity inhibition. In agreement with an unchanged secretion of fatty acids / triglycerides from hepatocytes into plasma in VLDL particles, relative mRNA expression levels of the VLDL synthesis genes apolipoprotein B and MTTP were not different between the two treatment groups (Figure 6C,D). Hepatic catabolism of fatty acids also did not appear to be affected by PRMT5 inhibition as judged from the null effect on gene expression levels of LIPE, ATGL and PNPLA3, PGC‐1alpha and PPARα, ACOX1, and CPT1 that mediate lipolysis and fatty acid oxidation, respectively (Figure 6E–K). In contrast, PRMT5 inhibition was associated with a significant increase in the expression of genes involved in fatty acid acquisition. More specifically, relative mRNA expression levels of the lipogenic transcription factor SREBF1 and the basolateral fatty acid transporter CD36 were respectively 59% higher (*p* < 0.05; Figure 6L) and 67% higher (*p* < 0.05; Figure 6M) in livers from GSK3326595‐treated mice than in those of controls. In addition, a significant increase in fatty acid synthase (FASN) transcript levels was detected in response to GSK3326595 treatment (+124%; *p* < 0.05; Figure 6N). PRMT5 inhibition did not impact relative mRNA expression levels of the lipogenic genes ACC and SCD1 (Figure 6O,P).

## 4 DISCUSSION

In the current study, we have tested, in a preclinical experimental setting, whether a causal relationship exists between PRMT5 activity and atherosclerosis development. We have observed that treatment with the PRMT5 inhibitor GSK3326595 shifts in vitro and ex vivo cultured macrophages to a pro‐inflammatory M1 phenotype in response to exposure to IFN‐gamma. However, no change in the basal macrophage and T cell activation state nor the susceptibility to atherosclerosis in Western‐type diet‐fed LDL receptor knockout mice was observed in response to GSK3326595 treatment.

We have shown that the PRMT5 inhibitor GSK3326595 is able to alter the macrophage phenotype (as evidenced by a change in the M1 and M2 marker gene expression pattern), particularly when IFN‐gamma is added. This is in line with the suggestion of Fan et al. that PRMT5 is a cellular sensor that connects stress stimuli (such as IFN‐gamma) to nuclear transcriptional events.^11^ The study of Fan et al. indicated that macrophages upon IFN‐gamma stimulation may induce PRMT5 translocation from the cytoplasm into the nucleus where PRMT5 binds to the promotor of the pro‐inflammatory gene MHCII to activate its transcription. In accordance, our in vitro study showed that PRMT5 inhibition by GSK3326595 in macrophages significantly downregulates the relative expression level of MHCII under IFN‐gamma stimulated conditions.

We found that GSK3326595 upregulates the expression level of the pro‐inflammatory marker IP‐10 upon IFN‐gamma stimulation, both in vitro and ex vivo. This is consistent with the study of Chai et al. in which the PRMT5 inhibitor EPZ015666 increased IP‐10 mRNA expression level as well as protein secretion in the human THP‐1 macrophage cell line.^4^ It is important to note though that the effect of PRMT5 on IP‐10 production depends on the cell type involved in the inflammatory response. In contrast to the inhibitory function of PRMT5 in macrophages, PRMT5 actually promotes TNF‐alpha‐induced IP‐10 production by endothelial cells.^19^

As IFN‐gamma is highly expressed in atherosclerotic lesions and has emerged as a significant factor in atherogenesis,^20^ we hypothesized that PRMT5 inhibition would result in an augmented immune response and increased atherosclerosis development in our atherosclerotic mouse model. Surprisingly, atherosclerosis susceptibility appeared unaltered upon treatment with GSK3326595. Our study also showed that the basal activation state of macrophages in the peritoneum was not changed by GSK3326595, nor were the T cell populations in several T cell‐rich compartments such as blood, spleen, and peritoneum. We therefore assume that the null effect on atherosclerosis susceptibility is due to the inability of GSK3326595 to alter the systemic inflammatory state. The fact that we did not observe any effect of PRMT5 inhibition on T cell activation is unexpected, since a previous study has indicated that blocking PRMT5 using the inhibitor CMP5^21^ severely suppressed the CD4^+^CD44^+^ memory T cell differentiation in the spleen of a murine model.^5^ In addition, T cell‐specific deletion of PRMT5 leads to peripheral T cell lymphopenia.^14^ We chose 5 mg/kg as our treatment dosage since Gerhart et al.^22^ showed that a dosage of 4.2 mg/kg GSK3326595 is sufficient to reduce SDMA production by >80% in vivo. However, it has been found that a daily dose of >50 mg/kg is required to inhibit PRMT5‐mediated cancer growth in mice.^22^ As such, it can be hypothesized that our 10‐fold lower dose is also too low to functionally inhibit PRMT5 in leukocytes, leading to the overall unchanged inflammation state after chronic GSK3326595 treatment.

Even though the inflammation state was not affected, the low‐dose GSK3326595 treatment for 9 weeks did cause a pharmacological effect in liver. More specifically, GSK3326595 treatment increased hepatic triglyceride accumulation in our LDL receptor knockout mice in response to Western‐type diet feeding. A common risk factor for hepatic steatosis and cardiovascular disease is hypertriglyceridemia.^23^ However, we did not find any increase in plasma triglycerides nor differences in atherosclerosis susceptibility, indicating that the effects on hepatic triglycerides are likely due to a local effect in liver. A previous study using AML12 hepatocytes found opposite results and showed that PRMT5 inhibition by EPZ015666 significantly reduced the lipid accumulation in this hepatocyte cell line.^18^ Huang et al. suggested that PRMT5 plays a role in mitochondrial biogenesis and fatty acid β‐oxidation and regulates liver metabolism through upregulating PPARα and PGC‐1 mRNA and protein expression.^18^ However, our gene expression measurements in livers of the Western‐type diet‐fed LDL receptor knockout mice showed only upregulated expression levels of CD36, involved in fatty acid uptake, and the hepatic lipogenic genes SREBF1 and FASN. We therefore anticipate that GSK3326595‐induced PRMT5 inhibition in our experimental setup modulated hepatic lipid metabolism via a different mechanism.

Important to note is that increased CD36 and FASN expression has shown to be related to increased hepatic steatosis.^24^ Hepatic steatosis arises from an imbalance between acquisition by uptake of fatty acids and de novo lipogenesis and triglyceride removal by fatty acid oxidation and the secretion of triglyceride‐rich lipoproteins such as VLDL.^24^ As CD36 and FASN are both involved in these processes, the upregulation of these genes could contribute to the observed increase in liver triglyceride levels.

In addition to the increased expression of CD36 and FASN, defective methylation of small heterodimer partner (SHP) leads to a higher hepatic triglyceride level as well as upregulated lipogenic gene expression.^25^ SHP has been identified as a PRMT5 methylation target, since the activity of SHP is increased by posttranslational methylation at Arg‐57 by PRMT5.^25^ Because the findings of Kanamaluru et al. are in line with our results, it can be speculated that the increased hepatic triglyceride level in our GSK3326595‐treated mice is possibly due to the inhibition of PRMT5‐depedent SHP methylation. However, further mechanistic studies are warranted to verify this hypothesis.

In conclusion, we have shown that PRMT5 inhibition by chronic low dose GSK3326595 treatment does not affect atherosclerosis development and lesion composition in Western‐type diet‐fed LDL receptor knockout mice, while it does induce hepatic triglyceride accumulation. Notably, GSK3326595 is currently being tested as anti‐cancer drug therapy in both preclinical mice studies and clinical phase I/II studies.^26^, ^27^, ^28^ In light of our current findings, it will be important to consider that long‐term pharmacological inhibition of PRMT5 to treat cancer may cause severe side effects in liver, i.e. induce non‐alcoholic fatty liver disease.

## AUTHOR CONTRIBUTIONS

**Yiheng Zhang:** Conceptualization (equal); data curation (equal); formal analysis (equal); investigation (equal); validation (equal); visualization (equal); writing – original draft (equal). **Robin A. F. Verwilligen:** Conceptualization (equal); data curation (equal); formal analysis (equal); investigation (equal); validation (equal); visualization (equal); writing – original draft (equal). **Miranda Van Eck:** Funding acquisition (lead); project administration (equal); supervision (equal); writing – review and editing (equal). **Menno Hoekstra:** Conceptualization (equal); methodology (equal); project administration (equal); supervision (equal); writing – review and editing (equal).

## FUNDING INFORMATION

This study was supported by VICI grant 91,813,603 from the Netherlands Organization for Scientific Research awarded to Miranda Van Eck. Miranda Van Eck is head of the Cardiovascular and Metabolic Therapeutics group (https://www.universiteitleiden.nl/en/science/drug‐research/biotherapeutics/cardiovascular‐and‐metabolic‐therapeutics) and an Established Investigator of the Dutch Heart Foundation (2007T056). Yiheng Zhang was supported by the Chinese Scholarship Council (CSC).

## CONFLICT OF INTEREST STATEMENT

The authors confirm that there are no known conflicts of interest associated with this publication and there has been no significant financial support for this work that could have influenced its outcome.

## Supporting information


Appendix S1.

## Data Availability

The data that support the findings of this study are available from the corresponding author upon reasonable request.
