# Supplementary material for: PRMT5 inhibition induces pro‐inflammatory macrophage polarization and increased hepatic triglyceride levels without affecting atherosclerosis in mice
Source: J Cell Mol Med. 2023 Mar 22;27(8):1056–68. doi: 10.1111/jcmm.17676 (PMC10098290; doi:10.1111/jcmm.17676)

Supplemental Figure 1: Gating strategy. Flow cytometry gating strategy for CD4 and CD8 cells in (A) blood, (B) peritoneal cells and (C) spleen.

Supplemental Material and methods

Gene expression analysis through real-time quantitative PCR

Cells dissolved in Guanidine Thiocyanate (GTC) (18.7g GTC (L-15809 Fisher Scientific), 0.3 g Sodium Citrate-dihydrate and 0.2 g n-Lauroyl Sarcosine in 50mL MilliQ) were used for RTqPCR analysis. Samples were stored in the -20 freezer until usage. To the GTC mixture, 50µl 2M NaAc (pH 5), 500µL water saturated Phenol and 200µl choroform was added and vortexed until a homogeneous yellow/white mixture. The mixture was centrifuged for 15 min at 12000 RCF 4 oC. After centrifugation, the upper water phase was transferred to a new 1.5ml tube without disturbing the inter-phase. Subsequently, 500µl isopropanol was added and centrifuged at maximal speed for 30 minutes at 4 oC (21382 RCF). After centrifugation, the supernatant is removed and the pellet is washed with 500uL 75% EtOH in MilliQ. After this, the samples were centrifuged at max speed (21382 RCF) for 5 minutes at 4 oC and the supernatant removed. After the pellet was dried, the pellet was dissolved in 20-50 µl MilliQ/5x106 cells or 100-200 µl MilliQ/100 mg organ.

The RNA concentration of the samples was measured using a nanodrop. From the RNA, cDNA was synthesized using reverse transcriptase (RT) reaction. In a clean 0.5mL tube 0.5-1ug (µl) total RNA was added and filled up to 12.4µl MilliQ. Subsequently, 1 µl oligo dT18 (100uM) and 0.5 ul random 9-mer (100uM) is added and placed in a PCR machine (Applied Biosystems 2720 Thermal Cycler)(PCR program: 5 min 65°C; 10min 25°C; 20min 50°C: 5min 85°C). After 5 minutes at 65°C, the tubes are directly placed on ice. 6.1µl reaction mix consisting of 4µl 5x RT buffer, 2µl 10 mM dNTP's, 0.1µl Maxima H Minus reverse transcriptase (200 U/µl) was added to the samples. For the negative control samples. instead of Maxima H Minus reverse transcriptase, 0.1µl MilliQ was added. After mixing (vortex briefly, spin down), the samples were placed back in the PCR machine and the PCR program was continued. After completion of the program, the samples were removed and 280/380µl of MilliQ water (for 0.5 µg/1µg RNA input) was added.

For Peer Review

For measuring gene expression, 4µl cDNA was pipetted at the bottom of a 96-well optical density plate. Per gene of interest a separated PCR mix (6µl/sample) (1.5µl SensiMix SYBR low-ROX, 0.15 µL primermix forward and reverse primers (15µM work-solution), 0.5µl QPCR buffer, 0.2µl 50mM $MgCl_2$ and 3.65µl MilliQ) was added to the cDNA. The plate was sealed with an optical adhesive cover and put in the RTqPCR machine.

For Peer Review

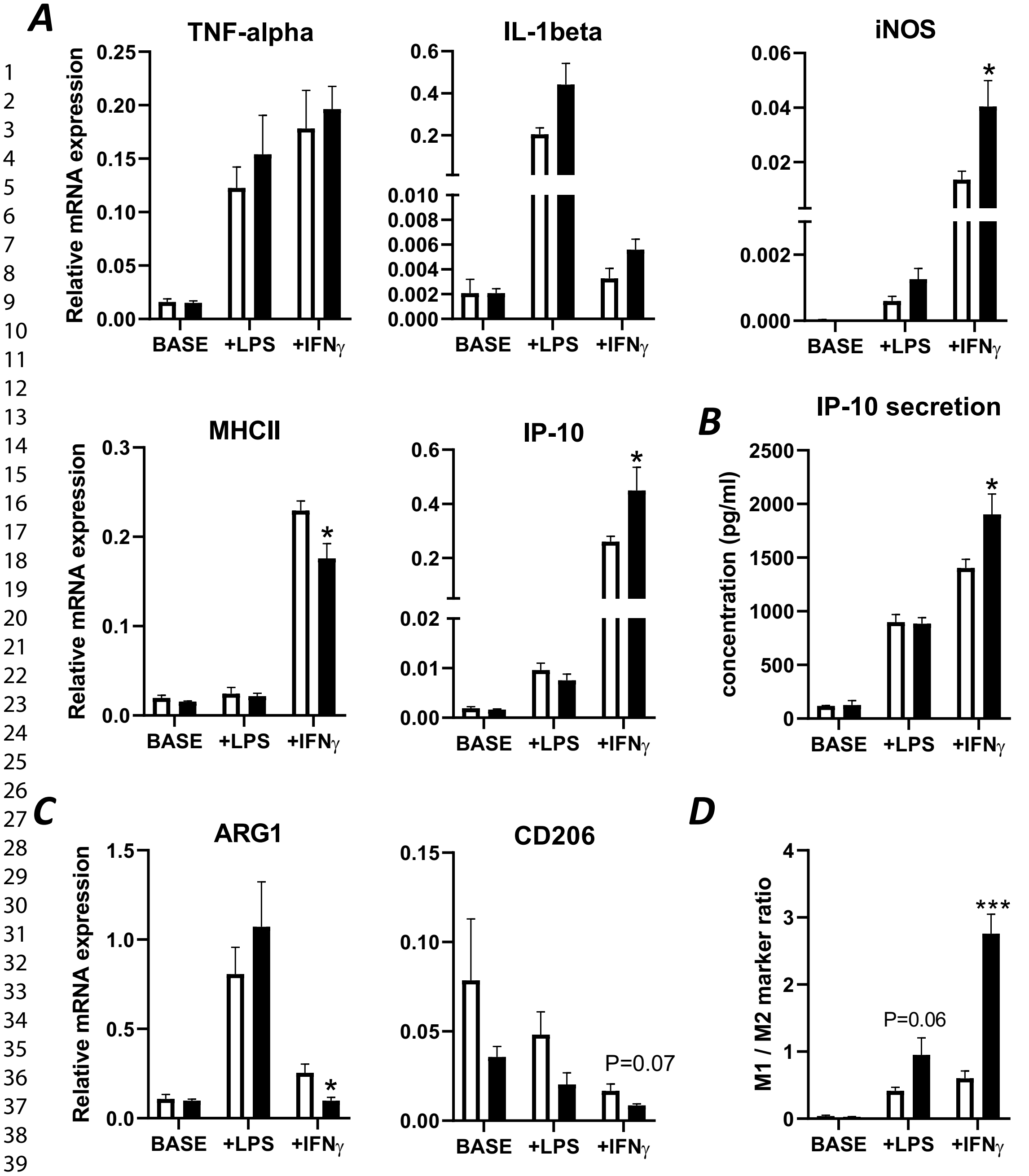
A
TNF-alpha
IL-1beta
iNOS
Relative mRNA expression
BASE
+LPS
+IFNγ
*
MHCII
IP-10
B
IP-10 secretion
concentration (pg/ml)
C
ARG1
CD206
P=0.07
D
M1 / M2 marker ratio
P=0.06
***

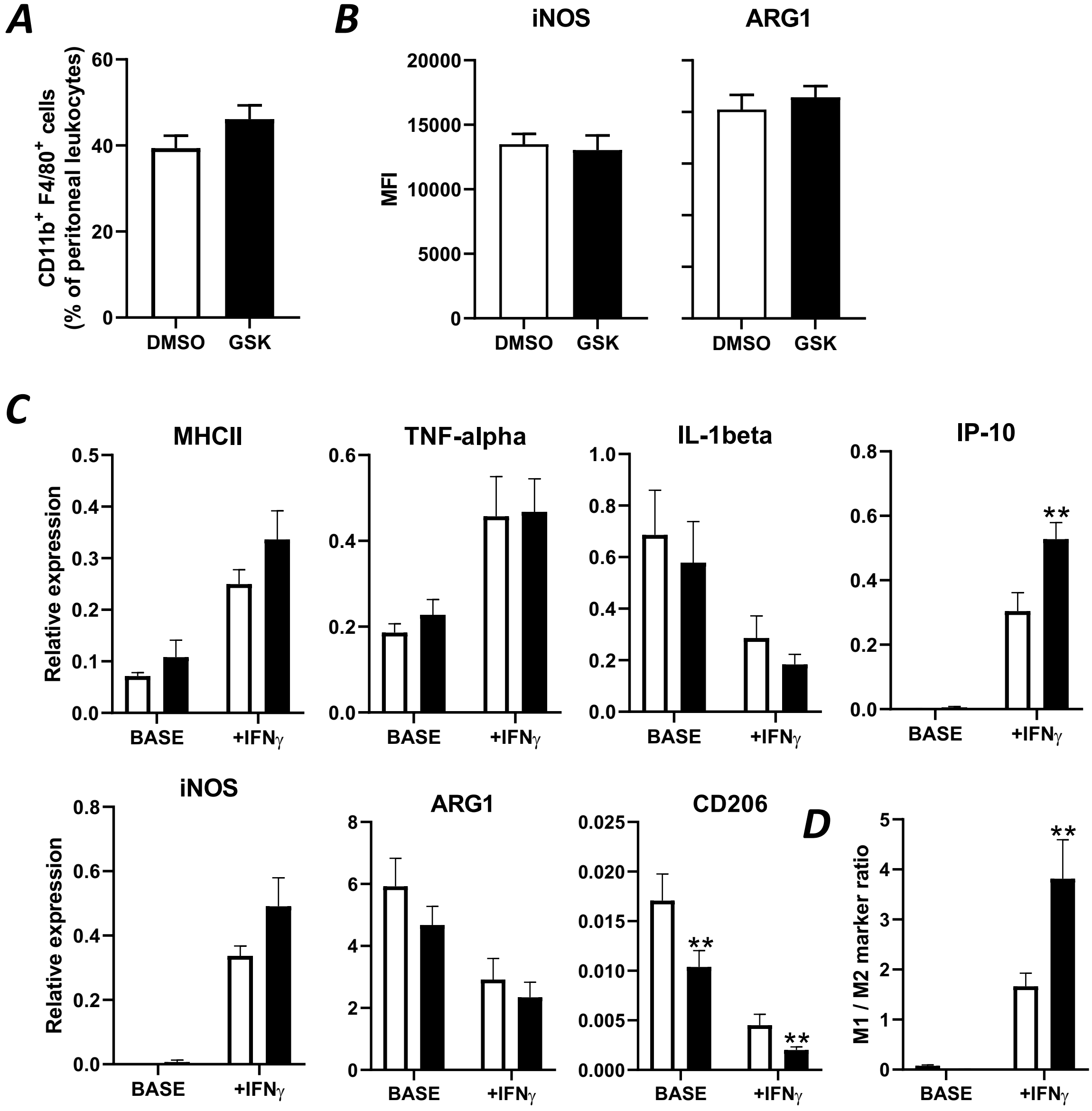
A
B
iNOS
ARG1
CD11b+ F4/80+ cells (% of peritoneal leukocytes)
MFI
DMSO
GSK
C
MHCII
TNF-alpha
IL-1beta
IP-10
iNOS
ARG1
CD206
D
Relative expression
M1 / M2 marker ratio
BASE
+IFNγ
**

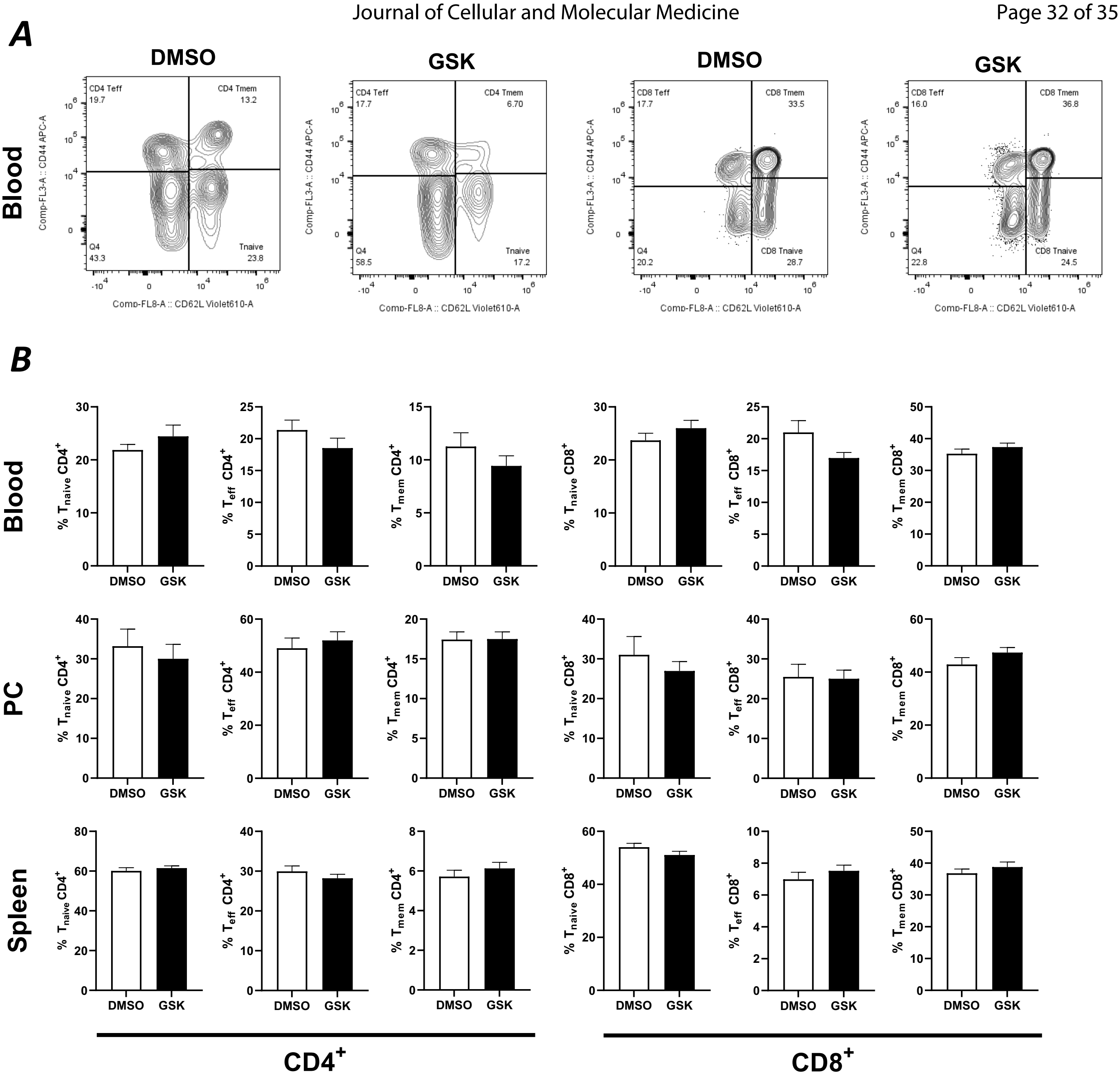
A
DMSO
GSK
DMSO
GSK
Blood
CD4 Teff
CD4 Tmem
Tnaive
Q4
CD8 Teff
CD8 Tmem
CD8 Tnaive
Comp-FL3-A :: CD44 APC-A
Comp-FL8-A :: CD62L Violet610-A
B
Blood
PC
Spleen
DMSO
GSK
CD4+
CD8+

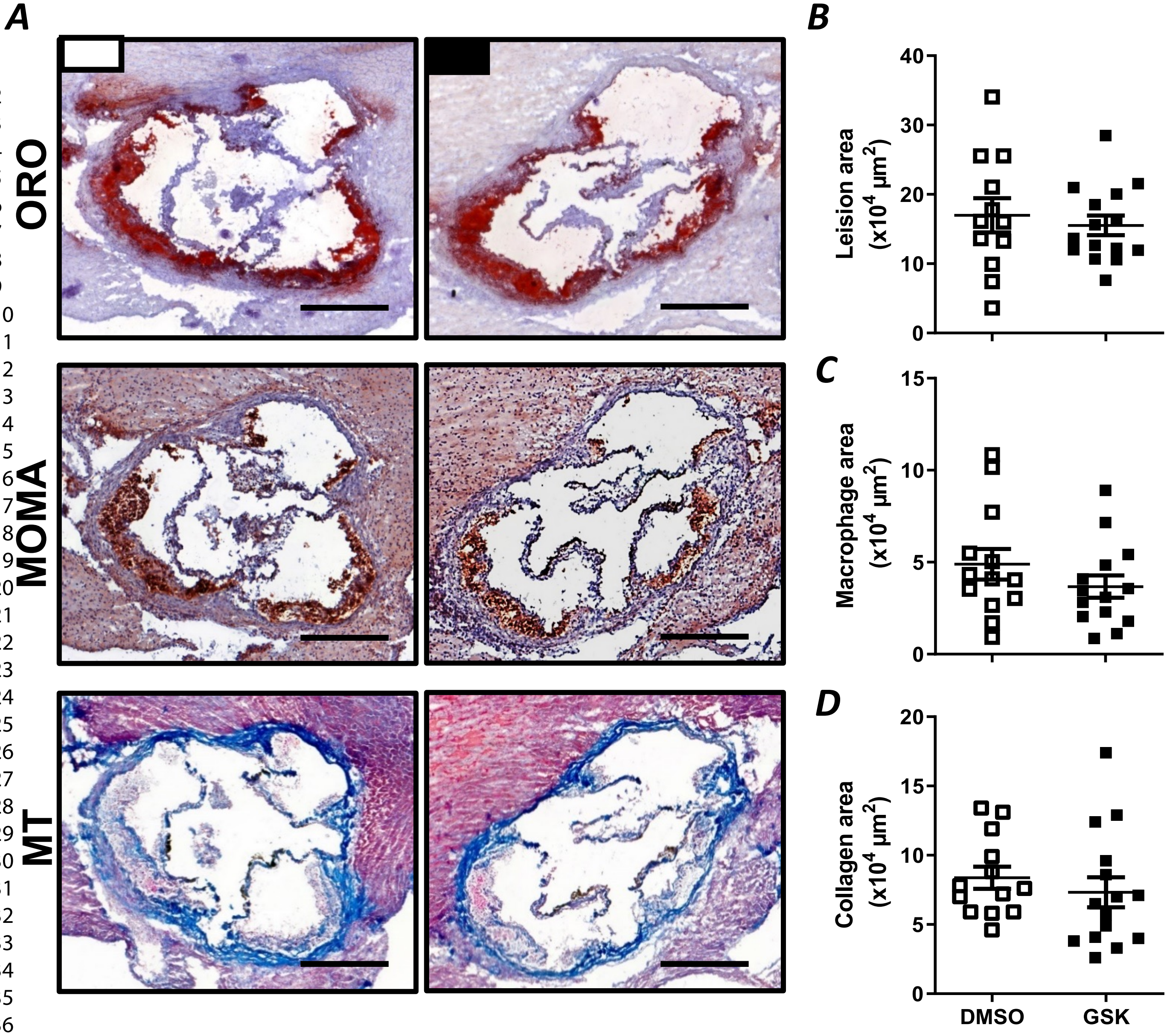
A
ORO
MOMA
MT
B
Leision area (x10^4 µm^2)
C
Macrophage area (x10^4 µm^2)
D
Collagen area (x10^4 µm^2)
DMSO
GSK

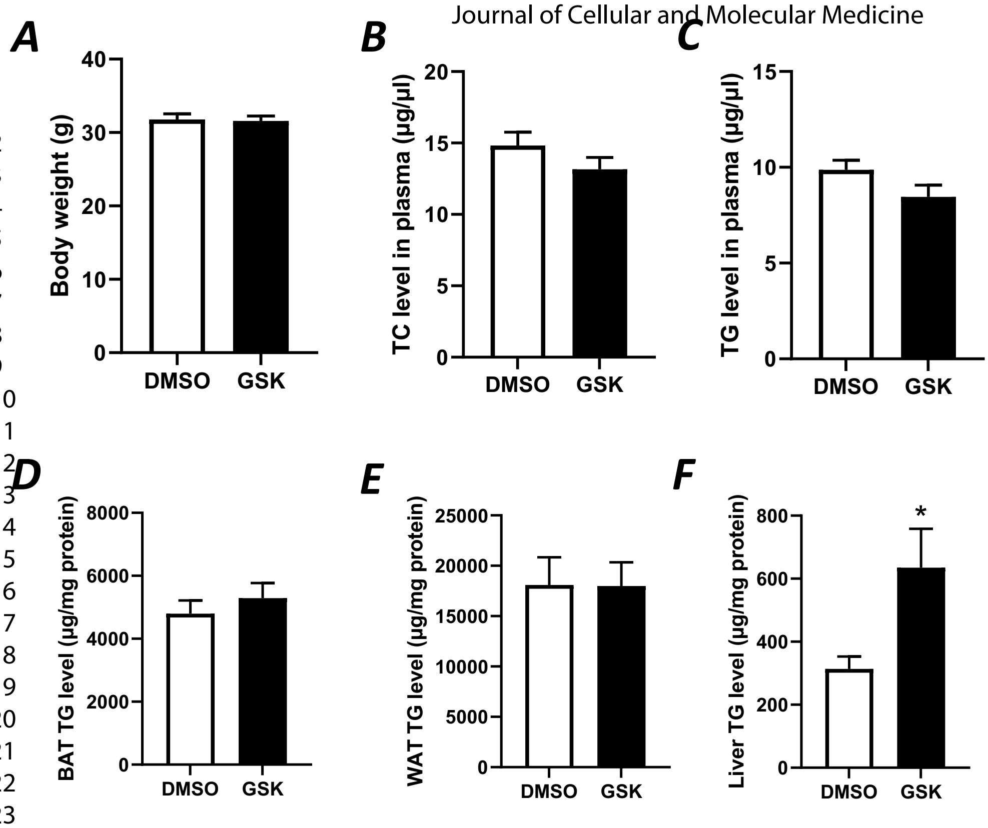
A
Body weight (g)
40
30
20
10
0
DMSO
GSK
B
TC level in plasma (µg/µl)
20
15
10
5
0
DMSO
GSK
C
TG level in plasma (µg/µl)
15
10
5
0
DMSO
GSK
D
BAT TG level (µg/mg protein)
8000
6000
4000
2000
0
DMSO
GSK
E
WAT TG level (µg/mg protein)
25000
20000
15000
10000
5000
0
DMSO
GSK
F
Liver TG level (µg/mg protein)
800
600
400
200
0
*
DMSO
GSK

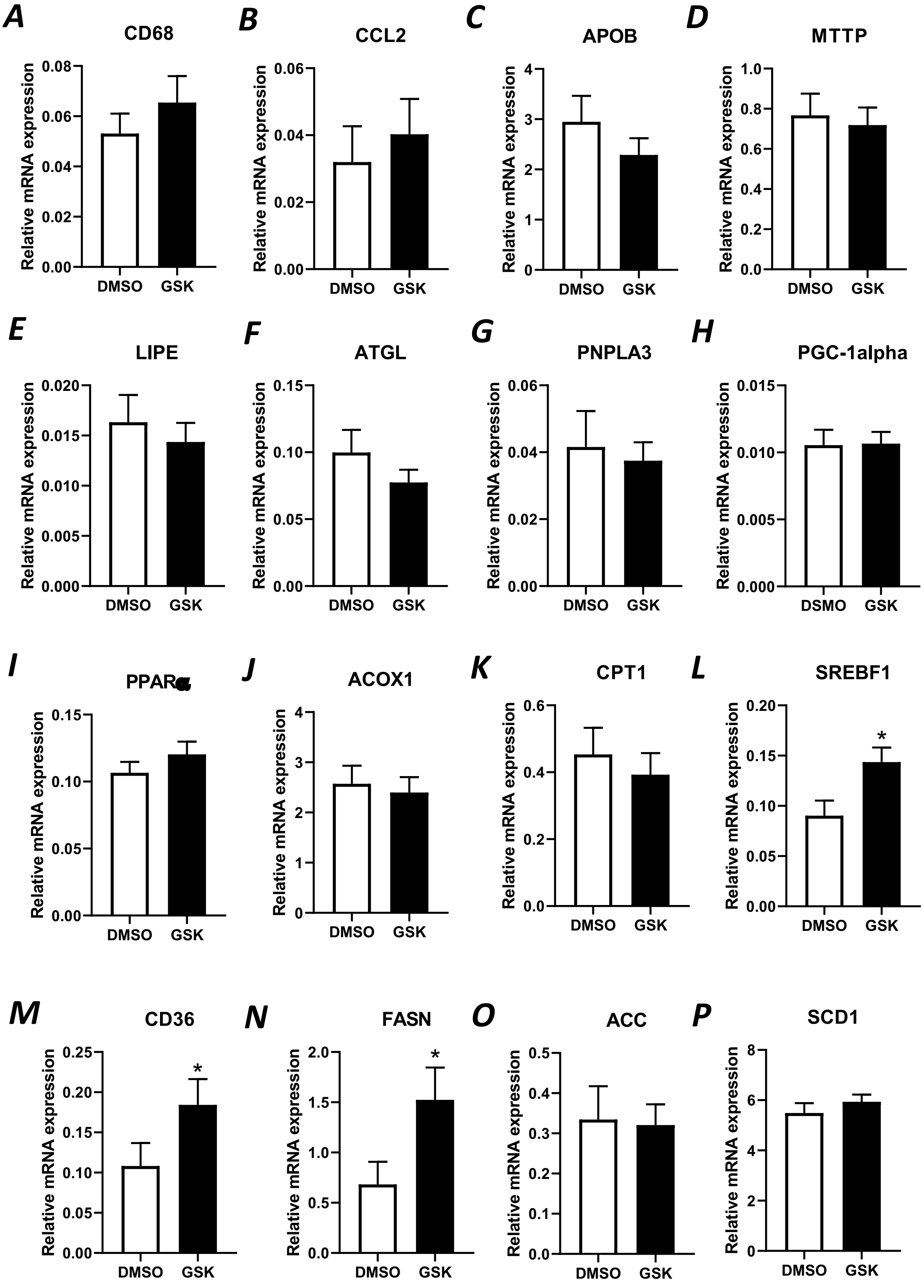
A
CD68
B
CCL2
C
APOB
D
MTTP
E
LIPE
F
ATGL
G
PNPLA3
H
PGC-1alpha
I
PPARα
J
ACOX1
K
CPT1
L
SREBF1
M
CD36
N
FASN
O
ACC
P
SCD1
Relative mRNA expression
DMSO
GSK
DSMO
*

Supplemental Figure 1

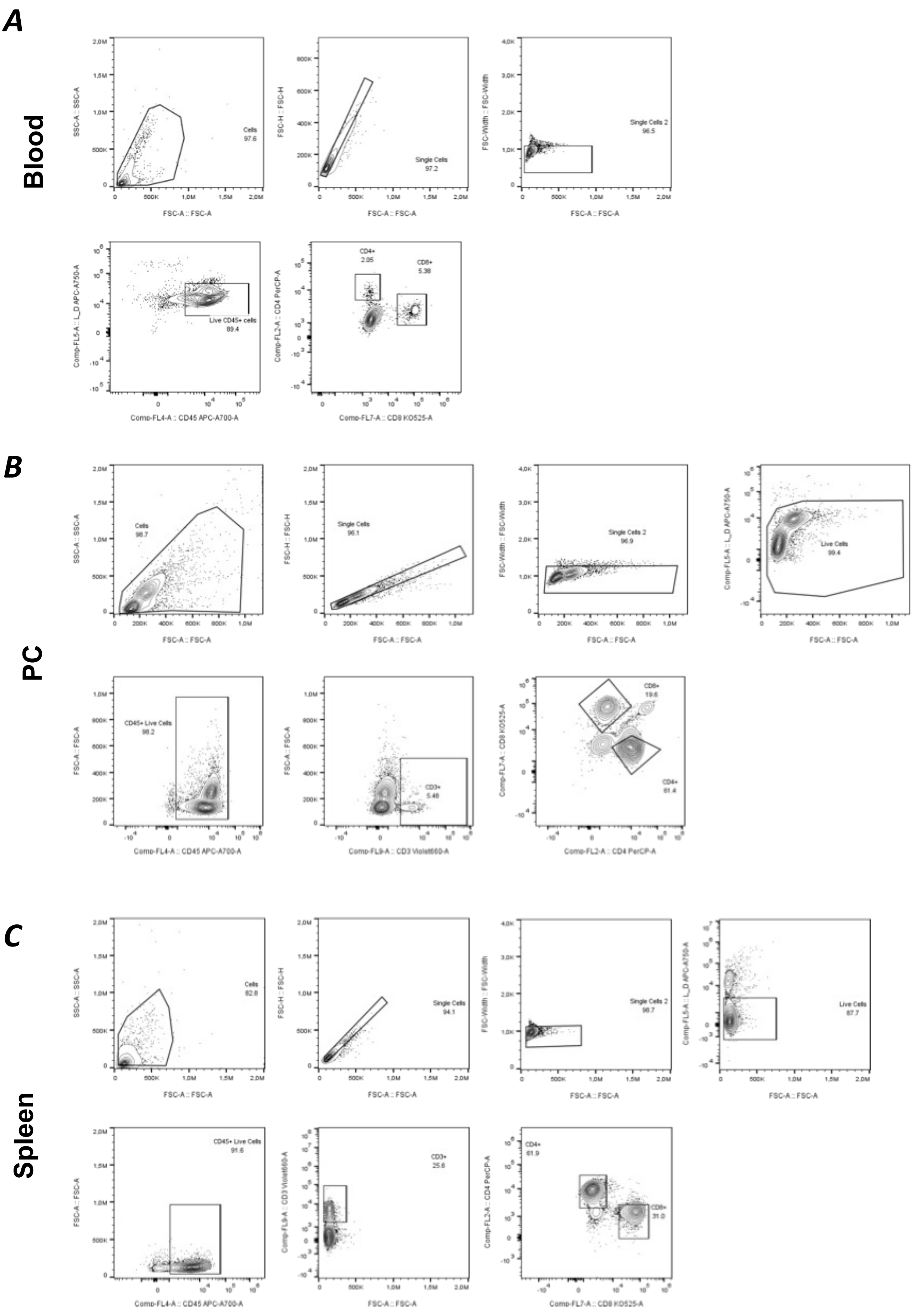

Supplement: Supplementary file 1 — Appendix S1. [file JCMM-27-1056-s001.zip › JCMM_17676-Supplementary.pdf]
