# Supplementary material for: PRMT5 inhibition induces pro‐inflammatory macrophage polarization and increased hepatic triglyceride levels without affecting atherosclerosis in mice
Source: J Cell Mol Med. 2023 Mar 22;27(8):1056–68. doi: 10.1111/jcmm.17676 (PMC10098290; doi:10.1111/jcmm.17676)
Supplement: Supplementary file 1 — Appendix S1. [file JCMM-27-1056-s001.zip › Supinfo.docx]

**Supplemental Figure 1: Gating strategy.** Flow cytometry gating strategy for CD4 and CD8 cells in (A) blood, (B) peritoneal cells and (C) spleen.

**Supplemental Material and methods**

**Gene expression analysis through real-time quantitative PCR**

Cells dissolved in Guanidine Thiocyanate (GTC) (18.7g GTC (L-15809 Fisher Scientific), 0.3 g Sodium Citrate-dihydrate and 0.2 g n-Lauroyl Sarcosine in 50mL MilliQ) were used for RTqPCR analysis. Samples were stored in the -20 freezer until usage. To the GTC mixture, 50μl 2M NaAc (pH 5), 500μL water saturated Phenol and 200μl choroform was added and vortexed until a homogeneous yellow/white mixture. The mixture was centrifuged for 15 min at 12000 RCF 4 oC. After centrifugation, the upper water phase was transferred to a new 1.5ml tube without disturbing the inter-phase. Subsequently, 500μl isopropanol was added and centrifuged at maximal speed for 30 minutes at 4 oC (21382 RCF). After centrifugation, the supernatant is removed and the pellet is washed with 500uL 75% EtOH in MilliQ. After this, the samples were centrifuged at max speed (21382 RCF) for 5 minutes at 4 oC and the supernatant removed. After the pellet was dried, the pellet was dissolved in 20-50 μl MilliQ/5x106 cells or 100-200 μl MilliQ/100 mg organ.

The RNA concentration of the samples was measured using a nanodrop. From the RNA, cDNA was synthesized using reverse transcriptase (RT) reaction. In a clean 0.5mL tube 0.5-1ug (μl) total RNA was added and filled up to 12.4μl MilliQ. Subsequently, 1 μl oligo dT18 (100uM) and 0.5 ul random 9-mer (100uM) is added and placed in a PCR machine (Applied Biosystems 2720 Thermal Cycler)(PCR program: 5 min 65°C; 10min 25°C; 20min 50°C: 5min 85°C). After 5 minutes at 65°C, the tubes are directly placed on ice. 6.1μl reaction mix consisting of 4μl 5x RT buffer, 2μl 10 mM dNTP’s, 0.1μl Maxima H Minus reverse transcriptase (200 U/μl) was added to the samples. For the negative control samples. instead of Maxima H Minus reverse transcriptase, 0.1μl MilliQ was added. After mixing (vortex briefly, spin down), the samples were placed back in the PCR machine and the PCR program was continued. After completion of the program, the samples were removed and 280/380μl of MilliQ water (for 0.5 μg/1μg RNA input) was added.

For measuring gene expression, 4μl cDNA was pipetted at the bottom of a 96-well optical density plate. Per gene of interest a separated PCR mix (6μl/sample) (1.5μl SensiMix SYBR low-ROX, 0.15 μL primermix forward and reverse primers (15μM work-solution), 0.5μl QPCR buffer, 0.2μl 50mM MgCl2 and 3.65μl MilliQ) was added to the cDNA. The plate was sealed with an optical adhesive cover and put in the RTqPCR machine.
